# Effect of Heat-Assisted and Ultrasound-Assisted Extraction Methods on the Phenolic Profile and Biological Activity of *Thymus vulgaris* L. Extracts

**DOI:** 10.3390/antiox14050567

**Published:** 2025-05-09

**Authors:** Rafael Mascoloti Spréa, Cristina Caleja, Eliana Pereira, Márcio Carocho, José Pinela, Tiane C. Finimundy, Ricardo C. Calhelha, Marina Kostić, Marina Soković, Miguel A. Prieto, Joana S. Amaral, Lillian Barros

**Affiliations:** 1 CIMO, LA SusTEC, Instituto Politécnico de Bragança, Campus de Santa Apolónia, 5300-253 Bragança, Portugal; rafael.sprea@ipb.pt (R.M.S.); ccaleja@ipb.pt (C.C.); mcarocho@ipb.pt (M.C.); jose.pinela@iniav.pt (J.P.); tiane@ipb.pt (T.C.F.); calhelha@ipb.pt (R.C.C.); jamaral@ipb.pt (J.S.A.); lillian@ipb.pt (L.B.); 2 Nutrition and Food Group (NuFoG), Department of Analytical Chemistry and Food Science, Instituto de Agroecoloxía e Alimentación (IAA)-CITEXVI, Universidade de Vigo, 36310 Vigo, Spain; mprieto@uvigo.es; 3 National Institute for Agricultural and Veterinary Research (INIAV), I.P., Rua dos Lágidos, Lugar da Madalena, 4485-655 Porto, Portugal; 4 Institute for Biological Research “Siniša Stanković”-National Institute of Republic of Serbia, University of Belgrade, 11000 Belgrade, Serbia; marina.kostic@ibiss.bg.ac.rs (M.K.); mris@ibiss.bg.ac.rs (M.S.)

**Keywords:** thyme, bioactive compounds, heat-assisted extraction, ultrasound-assisted extraction, response surface methodology (RSM)

## Abstract

This study compares the phenolic composition and antioxidant activity of extracts obtained using heat-assisted extraction (HAE) and ultrasound-assisted extraction (UAE), both optimized through response surface methodology (RSM), with the primary goal of maximizing the extraction of bioactive compounds from *Thymus vulgaris* L. Eight phenolic compounds were identified in the extracts, with rosmarinic acid being the most abundant. Depending on the selected conditions in each optimization, the concentration of this compound varied from 2.04 to 72.6 mg/g of extract for HAE and 0.73 to 73.5 mg/g of extract for UAE. The optimal conditions for each method that yielded the highest rosmarinic acid levels were as follows: for HAE, 27% ethanol at 91 °C for 121 min; and for UAE, 60 min at 500 W using 50% ethanol. The UAE-produced extract demonstrated superior antioxidant performance, particularly in inhibiting the formation of thiobarbituric acid reactive substances and oxidative haemolysis. Additionally, the extracts obtained using both optimized methodologies exhibited cytotoxic activity against five tumor cell lines and showed significant bactericidal and fungicidal effects against six bacterial and fungal strains. Overall, UAE proved more efficient as it enables the production of rosmarinic-acid-enriched extracts in less time, making them suitable as natural ingredients for industrial applications.

## 1. Introduction

The scientific community has extensively studied aromatic and medicinal plants due to their notable bioactive properties [1]. In particular, the food industry has shown a growing interest in these plants as sources of natural compounds, as they offer potential applications as natural ingredients to replace synthetic additives [2].

The demand for natural bioactive compounds as alternatives to synthetic additives, particularly in the food, pharmaceutical, and cosmetic industries [3], has surged due to a growing consumer preference for products that are safer, more sustainable, and derived from natural sources [4]. Plants from the Lamiaceae family are especially valuable in this context, as they contain a diverse range of phytochemicals with demonstrated bioactive properties. Within this family, *Thymus vulgaris* L., commonly known as thyme, is highly valued for its aromatic and medicinal properties. This perennial herb is widely used in cooking to enhance the flavor of dishes such as soups, sauces, and roasted meats, as well as for its role in food preservation. In most parts of the world, thyme is considered one of the most valuable spices and preservatives in the food industry, but it has also been extensively used for its medicinal effects, including carminative, digestive, antispasmodic, anti-inflammatory, and expectorant properties [5].

The bioactive properties of *T. vulgaris* are well-documented, with several studies highlighting its antioxidant [6], anti-inflammatory [7], cytotoxic [8], and antimicrobial effects [9]. These properties make *T. vulgaris* a valuable candidate for health-promoting applications, and different extraction techniques have been employed to maximize the recovery of its bioactive compounds [10,11,12]. In particular, rosmarinic acid, the major phenolic compound in thyme extracts, is notable for its strong antioxidant, anti-inflammatory, and antimicrobial activities [13]. Therefore, given the functional relevance of rosmarinic acid, optimizing its extraction from thyme is a crucial step in enhancing the utility of this herb as a source of natural bioactive compounds. Such optimization not only maximizes the yield of rosmarinic acid obtained but also ensures the effectiveness of thyme-based extracts as valuable functional ingredients for diverse industrial applications. Rosmarinic acid is a well-known polyphenol with strong antioxidant, anti-inflammatory, antimicrobial, and neuroprotective properties, making it highly valuable for applications in the food, pharmaceutical, and cosmetic industries. Its potent free radical scavenging activity contributes to oxidative stress reduction, while its anti-inflammatory effects have been linked to the modulation of key inflammatory pathways [14]. Additionally, its antimicrobial activity enhances the shelf life and safety of food products, reinforcing its role as a natural preservative [15].

The choice of extraction methodology, solvent, processing time, and temperature, among other variables, directly affects the quantity and diversity of the extracted bioactive compounds [16]. Thus, some authors have resorted to mathematical models such as the Response Surface Methodology (RSM), which facilitates the identification of the optimal extraction parameters, thereby enhancing the yield of the compounds of interest [17]. While several studies have characterized the extracts obtained from *T. vulgaris* through various extraction techniques, few have utilized mathematical models to determine the optimal extraction conditions. Previous studies on the optimization of antioxidants from this species have mostly focused on methods such as Soxhlet and accelerated solvent extraction [18] or pressurized liquid extraction [19]. Other studies have examined individual parameters, such as solvent type in UAE [20] or time and temperature in pressurized water extraction [21], but did not use design of experiments or response surface methodology to optimize multiple factors. More recently, RSM has been used to optimize UAE, focusing on other *Thymus* species, namely *T. serpyllum* [22,23].

As emerging green extraction technologies, heat-assisted extraction (HAE) and ultrasound-assisted extraction (UAE) offer sustainable and efficient alternatives for the recovery of bioactive compounds from plant matrices. UAE promotes cell disruption through acoustic cavitation, enhancing mass transfer and improving extraction efficiency at lower temperatures, whereas HAE facilitates the solubilization and diffusion of bioactive compounds through controlled thermal energy. These methods have been increasingly applied to maximize the extraction of bioactive compounds while preserving their structural integrity and bioactivity. However, a comprehensive comparison of these techniques, particularly under optimized conditions determined by response surface methodology (RSM), remains limited in the literature.

From this perspective, the present work provides a novel and comprehensive comparison of heat-assisted (HAE) and ultrasound-assisted extraction (UAE) techniques for optimizing the recovery of phenolic compounds from *T. vulgaris* dry leaves. By varying key extraction factors such as time (*t*, min), ethanol solvent concentration in water (*S*, %), temperature (*T*, °C), and power (*P*, W), this work employed a three-factor Box–Behnken Design (BBD) coupled with RSM to identify the optimal extraction conditions for maximizing phenolic compound yields, particularly focusing on rosmarinic acid.

The extracts obtained using the optimum extraction conditions were further evaluated for their bioactive potential regarding their antioxidant, antiproliferative, anti-inflammatory, antimicrobial, and antifungal activities.

## 2. Materials and Methods

### 2.1. Sample Preparation

Commercial dry leaves of *T. vulgaris* were provided by Deifil (Póvoa de Lanhoso, Portugal) in May 2021. The leaves were collected from the same field during the vegetative growth phase, specifically at the stage of shoot and leaf development, and then dried at 30 °C in industrial driers. The samples were reduced to a fine powder (∼20 mesh) using a blender (model A327R1, Moulinex, Barcelona, Spain) and stored in a desiccator at room temperature (∼25 °C), protected from light until further analysis.

### 2.2. Extraction Techniques

#### 2.2.1. Heat-Assisted Extraction (HAE)

*T. vulgaris* dry leaves were extracted through HAE performed in a thermostatic water bath under continuous electromagnetic stirring (CIMAREC Magnetic Stirrer with a fixed agitation speed of 500 rpm, (Thermo Scientific, San Jose, CA, USA) using sealed vessels to avoid solvent evaporation. Approximately 0.6 g of dry leaves were placed in a beaker with 20 mL of different concentrations of a hydroalcoholic solution according to the experimental design, where different levels of *t* (5 to 60 min), *S* (0–100%), and *T* (25–100 °C) were combined.

#### 2.2.2. Ultrasound-Assisted-Extraction (UAE)

The extraction of dry leaves of *T. vulgaris* was performed using an ultrasonic system (ultrasonic homogenizer, model CY-500, Optic Ivymen System, Barcelona, Spain; 20 kHz frequency) equipped with a titanium probe. Approximately 1.5 g of sample was placed in a beaker with 50 mL of different concentrations of a hydroalcoholic solution according to the experimental design, where different levels of *t* (5 to 60 min), *S* (0–100%), and *P* (50–500 W) were combined. To avoid the temperature increase, the beaker was placed in ice during the extraction.

In both cases, HAE and UAE, the solid/liquid ratio was kept at 30 g/L. Finally, each extraction was filtered through a Whatman paper filter No. 4, and an aliquot of each point was filtered through disposable 0.22 µm filters for HPLC analysis.

### 2.3. Experimental Design

A three-factor Box–Behnken Design (BBD) coupled with response surface methodology (RSM) was implemented to optimize the extraction of phenolic compounds from the dry leaves of *T. vulgaris.*

The varying factors for HAE were *X*_1_ (*t*; min), *X*_2_ (*T*; °C), and *X*_3_ (*S*; %), and the variable intervals were set based on previously studies in the literature [22,24], which indicates that there are significant variations in the ideal set of conditions depending on the target compounds and the matrix characteristics. Thus, larger intervals were used to increase the flexibility of the model and to grasp the behavior of the system under different conditions. The central points are also used to understand the error associated with operator procedures. The experimental design allows to obtain robust response surface models, especially when the goal is to optimize a process rather than to identify significant factors.

Regarding UAE, the three varying factors were *X*_1_ (*t*; min), *X*_2_ (*P*; W), and X_3_ (*S*; %) which were also defined based on the available literature [20,25]. For both cases, data are shown in Table 1. Ethanol-based solvents were selected due to their ability to enhance the solubility of a broad range of polyphenols while ensuring food-grade safety and industrial applicability. The ethanol concentration ranges selected were based on previous studies demonstrating that intermediate ethanol–water mixtures enhance the extraction of both polar and moderately non-polar phenolic compounds.

### 2.4. Response Surface Methodology for Optimization Purposes

The BBD used three factors and repeated the center value (five times), resulting in 17 runs, which were randomly performed and analyzed. A second-order least squares polynomial equation was used to determine and fit the response superficies models.

(1) $$Y=b_{0}+\sum_{i=1}^{n}b_{i}X_{i}+\sum_{\begin{array}{c} i=1\\ j>i \end{array}}^{n-1}\sum_{j=2}^{n}b_{ij}X_{i}X_{j}+\sum_{i=1}^{n}b_{ii}X_{i}^{2}$$

where *Y* corresponds to the dependent variables or responses to be modeled, *X_i_* and *X_j_* define the independent variables, *b*_0_ is the constant coefficient, *b_i_* is the linear effect coefficient, *b_ij_* is the interaction effect coefficient, *b_ii_* is the quadratic effect coefficient, and n is the number of variables. The optimization was performed separately for each extraction method (UAE and HAE).

The responses analyzed were the dry weight value of the extraction yield residue (E, g dw) and rosmarinic acid yields (RA, mg RA/g dw E).

### 2.5. Analysis of the Relevant Responses

#### 2.5.1. Extraction Yield (Residue)

For the dry residue evaluation, 10 mL of each extract was dried in an oven at 100 ± 1°C until constant weight and the dry residue was measured. The content of rosmarinic acid was determined using HPLC-DAD-ESI-MS/MS, as described below.

#### 2.5.2. Determination of the Individual Profile of Phenolic Compounds

The individual profile of phenolic compounds was determined according to Bessada et al. [26]. Each experimental design point was lyophilized (Freezone 4.5, Labconco, Kansas City, MO, USA) and dissolved in an aqueous solution of ethanol (20% *v*/*v*) up to a final concentration of 10 mg/mL and filtered through disposable 0.22 µm filters. The analysis was made using Dionex Ultimate 3000 ultra-performance liquid chromatographic equipment (Dionex Ultimate 3000 UPLC and Linear Ion Trap LQT XL, Thermo Scientific, San Jose, CA, USA) equipped with a quaternary pump and a diode array coupled in series to an electrospray ionization mass spectrometry detector (HPLC-DAD-ESI/MS). Acetonitrile (99.9%) was of HPLC grade, and ethanol (98.8%) was of analytical grade from Fisher Scientific (Lisbon, Portugal). The phenolic compounds were identified by comparing their retention time, UV-vis, and mass spectra with those obtained from standard compounds or with the literature. For the compounds’ quantification, the following standards (Sigma-Aldrich, St. Louis, MO, EUA) were used to obtain the calibration curves: apigenin-6-*C*-glucoside (y = 225,142x + 112,350, R^2^ = 0.9989); caffeic acid (y = 47,221x − 228,640, R^2^ = 0.9989); catechin (y = 8387.1x − 71,124, R^2^ = 0.9964); rosmarinic acid (y = 25,054x − 171,281, R^2^ = 0.9975). In cases where the standards were not available, the compound was quantified using the most chemically similar standard. The results were expressed in mg per g of lyophilized extract.

### 2.6. Bioactivity Evaluation

#### 2.6.1. Antioxidant Potential of the Extracts

The antioxidant potential of the optimized extracts was evaluated through five distinct in vitro assays, each assessing different mechanisms of antioxidant action. The assays included the Thiobarbituric Acid Reactive Substances (TBARS) assay, which measures lipid peroxidation; the 2,2-diphenyl-1-picrylhydrazyl (DPPH) radical-scavenging assay, which determines free radical neutralization capability; the reducing power assay, which indicates the electron-donating capacity of the extract; the Cellular Antioxidant Activity (CAA) assay, which assesses intracellular antioxidant effects in a cell-based model as described by Spréa et al. [27]; and the Oxidative Haemolysis Inhibition Assay (OxHLIA), as previously detailed by Lockowandt et al. [28], which evaluates the extract’s ability to protect erythrocytes from oxidative damage induced by AAPH.

For each assay, results were expressed as EC_50_ or IC_50_ values (μg/mL), representing the extract concentration required to inhibit 50% of the oxidative or radical activity. Trolox, calcium ascorbate (E-302), and potassium metabisulfite (E-224) were used as positive controls across assays. For OxHLIA, results were expressed as IC_50_ values specific to Δt of 60 and 120 min, indicating the concentration necessary to protect 50% of erythrocytes from AAPH-induced haemolysis over these time intervals. In the CAA assay, results were presented as the percentage inhibition of the oxidation reaction, with quercetin as the positive control.

#### 2.6.2. Antiproliferative Activity in Tumor and Non-Tumor Cell Lines

The cytotoxic activity of the optimized *T. vulgaris* extract was evaluated according to the sulforhodamine B (SRB) colorimetric assay, as previously described by Abreu et al. [29]. The results were expressed as the extract concentration required to inhibit 50% of cell growth (GI_50_ value, μg/mL). Three human tumor cell lines were tested: AGS (human gastric epithelial cell line), CaCo-2 (human colorectal adenocarcinoma), and MCF-7 (breast carcinoma). Additionally, toxicity was assessed using VERO cells (kidney epithelial cells from the African green monkey), which were obtained from the Leibniz-Institute DSMZ-German Collection of Microorganisms and Cell Cultures GmbH. Ellipticine was used as a positive control.

#### 2.6.3. Anti-Inflammatory Activity

The anti-inflammatory potential of the optimized extracts was evaluated in RAW 264.7 murine macrophage cells (sourced from the European Collection of Authenticated Cell Cultures). The assay focused on measuring the inhibition of nitric oxide (NO) production induced by lipopolysaccharides (LPS), following the method described by [27]. The results were presented as the concentration of extract causing 50% inhibition of NO production (IC_50_, µg/mL). Dexamethasone was used as a positive control.

#### 2.6.4. Antimicrobial and Antifungal Activity

The evaluation of antimicrobial and antifungal assays were performed according to the protocol established by Ivanov et al. [30]. The antibacterial activity of the extracts was tested against a panel of Gram-positive and Gram-negative bacteria, including *Staphylococcus aureus* (ATCC 11632), *Bacillus cereus* (clinical isolate), *Listeria monocytogenes* (NCTC 7973), *Escherichia coli* (ATCC 25922), *Salmonella typhimurium* (ATCC 13311), and *Enterobacter cloacae* (ATCC 35030).

For the antifungal activity, six micromycetes were tested: *Aspergillus fumigatus* (human isolate), *Aspergillus niger* (ATCC 6275), *Aspergillus versicolor* (ATCC 11730), *Penicillium funiculosum* (ATCC 36839), *Trichoderma viride* (IAM 5061), and *Penicillium verrucosum var. cyclopium* (food isolate).

The results were expressed as minimal inhibitory concentration (MIC), minimal bactericidal concentration (MBC), and minimal fungicidal concentration (MFC), all in mg/mL.

### 2.7. Statistical Analysis

Analyses were performed in at least technical triplicate and results were expressed as mean ± standard deviation (SD). All bioactivity assays were performed on the same extract batch to ensure comparability of results. The optimization protocol was performed using Design Expert 11 (Stat-Ease, Minneapolis, MN, USA), based on BBD and RSM for the design of experiments and followed by optimization of the solid residue response. The experimental design included three factors and 17 independent combinations (runs), including five replicates of the center position.

Statistical analysis was performed using SPSS Statistics software (version 23, IBM, Chicago, IL, USA). The Shapiro–Wilk test was applied to assess the normality of the data, and Levene’s test was used to evaluate homoscedasticity (equal variance assumption). A Student’s t-test was conducted at a 95% confidence level (*p* < 0.05) to determine statistical significance between the optimized samples.

## 3. Results and Discussion

### 3.1. Optimization Analysis

#### Phenolic Compounds Identification

Total polyphenols were calculated as the sum of identified compounds determined using HPLC-DAD-ESI-MS/MS (Table S1). The study showed that the phenolic composition of the extracts is dependent on the extraction conditions, with substantial variations in the TPC and the relative proportion of the different compounds.

The phytochemical analysis revealed a profile including eight phenolic compounds, namely gallocatechin (P1), caffeic acid hexoside (P2), caffeic acid acetylhexoside (P3), apigenin-*C*-hexoside-*O*-hexoside (P4), rosmarinic acid hexoside (P5), luteolin-7-*O*-glucuronide (P6), *cis*-rosmarinic acid (P7), and luteolin-*O*-diglucuronide (P8). These compounds, which are recognized for their antioxidant and anti-inflammatory activities, contribute substantially to the biological activity of *T. vulgaris* extracts. Notably, rosmarinic acid (P7) emerged as the dominant compound, confirming the findings by Sprea et al. [31]. Table 2 provides the phenolic compounds identified in each run of both optimizations (HAE and UAE), with the TPC calculated as the sum of the major peaks identified.

The process optimization strategy was based on studying, selecting, and maximizing the yield of the extract and the yield of the main compound, rosmarinic acid. The extract yield (dry residue) was selected as a response variable because it is simple to determine, does not require specialized analytical equipment, and could be applied in an industrial setting. On the other hand, rosmarinic acid was prioritized during the optimization process as it is the major compound and possesses well-established bioactivity, making it highly relevant for applications in food, cosmetics, and nutraceuticals or dietary supplements. Moreover, industries frequently look for rosmarinic-acid-enriched extracts, particularly those with standardized contents of this compound. Consequently, rosmarinic acid represented the best parameter for evaluating the efficiency of the process.

All compounds were detected in at least one run of each technique, confirming the suitability of the chosen techniques and selected compounds. However, some compounds seem to be selectively extracted depending on the technique. The extraction with UAE seems to be more selective, since some compounds, such as P3 and P4, were barely detected and others, such as P5, P6 and P8, were not detected in specific conditions. Regarding the effect of each variable over compounds extraction, it can be noted that *P* (*X*_2_*^UAE^*) of extraction significantly influences the recovery of the polyphenols. For example, in runs 2, 9, and 11 (*X*_2_ = 500 W), TPCs of 32.6, 123.0 and 33.0 mg/g E are achieved, respectively, which is an improvement over the lower power conditions. This was particularly evident in P7, whose extraction ranged from 0.7 to 73.5 mg/g E, in case of the lowest and maximum *P* (*X*_2_), respectively. Regarding T (*X*_2_*^HAE^*), higher temperatures favored the extraction of compounds when the solvent used was appropriate. For example, in runs 2 and 9 with *X*_2_ = 100 °C, the maximum TPCs were obtained (152.3 and 127.7 mg/g E, respectively), indicating that the TPC is higher under these conditions. Consequently, in runs 4 and 10 with *X*_2_ = 25 °C, the TPC decreased significantly (54.8 and 12.2 mg/g E, respectively). This confirms the importance of the *T*. As for the interaction between variables, it was noted that as the *T/P* (*X*_2_) and the extraction time *t* (*X*_1_) increased, the TPC and the number of extracted compounds also increased. This is clearly observed in run 9 (*X*_1_ = 180 min), where TPC and individual compounds show a significant increase compared to runs with a lower extraction time, such as run 7, with *X*_1_ = 5 min. *S* (*X*_3_) also had a significant impact, since its decrease improves ultrasonic cavitation in low-viscosity and high-vapor-tension media and enhances the recovery of phenolics in UAE. However, *S* (*X*_3_) is not as influential as in UAE, as heat facilitates the diffusion of polyphenols regardless of the proportion of EtOH.

The optimization was based on the balance between the yield and the efficiency of the process, giving priority to the recovery of the predominant compound. UAE and HAE operated at different *T* and *p*, yet degradation compounds were not detected in the chromatograms, although minor changes in the relative abundance of specific compounds were found, especially at higher *T* conditions in HAE.

Thanks to the RSM experimental design, optimal conditions could be established to maximize the recovery of rosmarinic acid (RA). These results showed that UAE was superior to HAE in terms of efficiency, achieving the highest yields of rosmarinic acid in a shorter time. Under optimal conditions (*X*_1_ = 60 min; *X*_2_ = 500 W; *X*_3_ = 50%), UAE reached 73.6 mg RA/g E and a maximum TPC of 123.0 mg/g E. In contrast, the extraction required less use of solvent (*X*_3_ = 27%). For HAE, the increase of *T* (91 °C) and *t* (121 min) improved the recovery of the compounds, achieving a similar overall efficiency of the process (124.2 mg/g E), while the recovery of the major compound was slightly lower (72.0 mg RA/g E) compared to UAE.

The selection of UAE and HAE was based on their efficiency, cost-effectiveness, and industrial applicability for the extraction of bioactive compounds from *T. vulgaris.* These methods are widely recognized for effectively extracting and preserving phenolic compounds and essential oils, while requiring simpler equipment and lower operating costs compared to more advanced techniques. Their scalability and wide use in the food and pharmaceutical industries further support their selection. To prioritize the optimization of rosmarinic acid, the parameters for HAE and UAE were determined using the BBD with specific settings for *t*, *P*/*T*, and *S*.

Regarding the optimization of the two extraction methods via RSM, 17 individual runs were conducted for each (Table 2), and two responses were selected. *R_HAE_* and *RA_HAE_* represented the dry residue (extraction yield) and the rosmarinic acid content, respectively (Figures S1 and S2).

In the case of HAE, for *R_HAE_* the quantities ranged from 0.0026 to 0.0232 g. The obtained model was significant, although linear, and showed a *p*-value of 0.0029 with one ignored value (outlier) and the following Equation (2):

(2) $$R_{HAE}=-0.001634+0.000033x_{1}+0.000180x_{2}-0.000038x_{3}$$

The R^2^ was 0.67, while the predicted R^2^ was adjusted to 0.4738, which indicates suboptimal model quality. Due to the linear nature of the model, it exhibited limited interactions among the factors, resulting in an unsatisfactory fit for the response “dry residue” ($R_{HAE})$. Alternative models, such as quadratic or interaction-based models, were considered but did not significantly improve the fit without increasing model complexity and reducing interpretability. Considering the limited predictive capacity of this model, only the optimization towards rosmarinic acid was considered.

Among the 17 runs, the amounts of rosmarinic acid varied between 2.04 and 72.63 mg/ g E. The model showed a quadratic equation with a significant *p*-value of 0.0024 and a non-significant lack of fit. The R^2^ was set at 0.9328 and the predicted R^2^ was 0.8464. Using the maximize function, the predicted amount of rosmarinic acid was 72.1 mg/ g E, with an optimal point corresponding to 121 min of extraction time, at 91 °C, using 27% ethanol. The resultant Equation (3) of the optimization was as follows:

(3) $${RA}_{HAE}=+\;54.92+9.630x_{1}+17.76x_{2}-8x_{3}+11.93x_{1}x_{2}-4.01x_{1}x_{3}-18.41x_{2}x_{3}-22.47x_{1}^{2}-6.83x_{2}^{2}-24.16x_{3}^{2}$$

**Figure 1:**
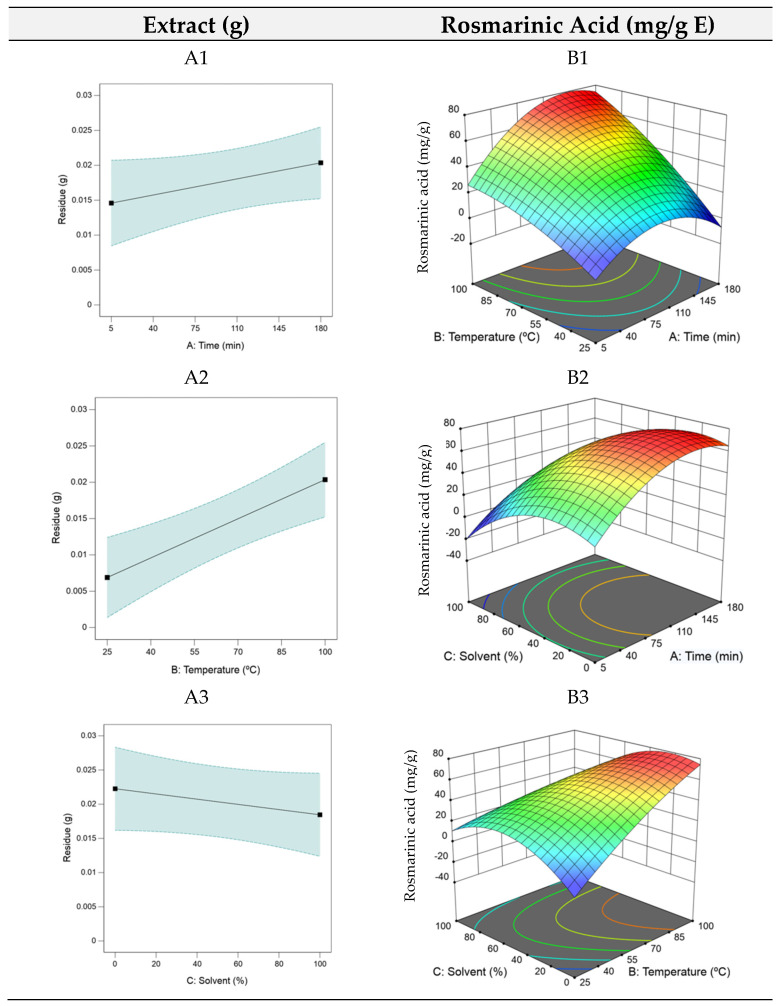
3D plots of the dry residue (*R_HAE_*) and rosmarinic acid (*RA_HAE_*) for heat–assisted extraction (HAE). Plots (**A1**–**A3**) represent the dry residue yield (RHAE), and plots (**B1**–**B3**) show the rosmarinic acid content (RAHAE, mg/g extract), evaluated as functions of extraction time (A), temperature (B), and ethanol concentration in the solvent (C).

Analysing Equation (3) reveals that *t* and *T* ($x_{1}$ and $x_{2}$) had the most significant impact on rosmarinic acid yield, while high *S* (*X*_3_) exerted a negative influence, hence the low ethanol level at the optimal point. The RSM plots for HAE are shown in Figure 1. As the model for the optimization of the residue yield was only linear, each factor required individual analysis (A1, A2, and A3).

The RSM plots indicated that the dry residue increased with longer extraction time and higher temperatures but decreased as the ethanol percentage increased. Despite this increase, the dry residue likely contains a range of compounds, many of which may lack bioactive properties. This characteristic suggests that the dry residue, which tends to accumulate over prolonged extraction times, is not a reliable predictor of an extract’s bioactivity. Consequently, the optimization focused on maximizing the rosmarinic acid content due to its diverse range of biological activities and potential applications, both for health applications and functional foods formulation. For rosmarinic acid optimization, as seen in the B1, B2, and B3 plots in Figure 1, intermediate extraction times facilitated optimal compound extraction, with higher temperatures (B1) enhancing this effect. Lower ethanol quantities proved favorable (B3), supporting efficient extraction of bioactive components.

Regarding UAE, the responses for *R_UAE_* (dry residue) varied between 0.003 and 0.02 g among the 17 runs. The quadratic model obtained was significant, showing a *p*-value of 0.0052 and a non-significant lack of fit. The model demonstrated a R^2^ of 0.9153 and predicted R^2^ of 0.3846. The equation of the model was

(4) $$R_{UAE}=+\;0.0049+0.0025x_{1}+0.0054x_{2}-0.0011x_{3}+0.0015x_{1}x_{2}-0.00x_{1}x_{3}+0.0006x_{2}x_{3}+0.0011x_{1}^{2}+0.0039x_{2}^{2}+0.00x_{3}^{2}$$

**Figure 2:**
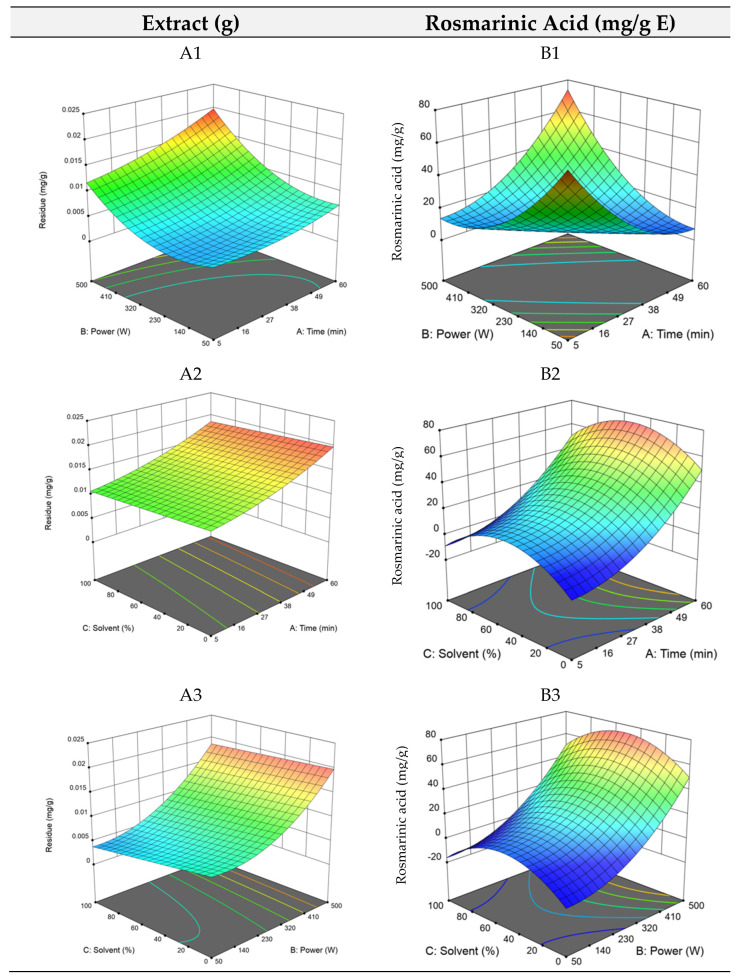
3D plots of the dry residue (*R_HAE_*) and rosmarinic acid (*RA_HAE_*) for ultrasound–assisted extraction. Plots (**A1**–**A3**) represent the dry residue yield (RHAE), and plots (**B1**–**B3**) show the rosmarinic acid content (RAHAE, mg/g extract), evaluated as functions of extraction time (A), ultrasound power (B), and ethanol concentration in the solvent (C).

The RSM plots are shown in Figure 2, where longer extraction times and high ultrasound power (A1) and low ethanol quantities (A2) promote the extraction of compounds. Overall, once again the model for maximizing the dry residue (total extraction yield) did not show high robustness, and thus, the focus was once again given to the optimization of rosmarinic acid extraction.

Concerning the UAE extraction of rosmarinic acid, a significant quadratic model was obtained with a *p*-value of <0.0001 and a non-significant lack of fit, indicating a well-fitting model. The R^2^ and predicted R^2^ values were 0.9936 and 0.9893, respectively, demonstrating a much more robust model compared to that for the optimization of the dry residue yield. However, one outlier, run 12, was excluded from the model due to its deviation from the general trend since several compounds were not detected. By removing this outlier, the model improved its R^2^ and showed a non-significant lack of fit, improving the overall results. Overall, one extraction point, run 9, showed a drastic increase in the amount of rosmarinic acid, several magnitudes higher than the other runs, which clearly influenced the model. The quadratic equation that defined the optimization is

(5) $${RA}_{UAE}=9.2-0.5613x_{1}+2.77x_{2}-0.0240x_{3}+30.42x_{1}x_{2}+0.0027x_{1}x_{3}+0.2519x_{2}x_{3}+15.02x_{1}^{2}+16.70x_{2}^{2}-23.05x_{3}^{2}$$

By analysing the equation, it is evident that power and solvent concentration were the most influential factors, while extraction time had a comparatively minor effect. As expected, the interaction between time and energy was the most important combination of factors affecting the optimal points, which, as stated above, was highly influenced by run 9. The optimal point was established at 60 min of extraction time, 500 W of ultrasound power, and 50% of ethanol, previewed to yield 73.4 mg/g E of rosmarinic acid. The RSM plots for UAE (Figure 2, plots B1, B2, and B3) illustrate the pronounced effect of time and ultrasound power, particularly in plots B2 and B3. In B3, the middle values of the solvent can be seen by the hump in blue on the left side of the plot. Overall, as for HAE, optimizing the amounts of rosmarinic acid in UAE proved to be more reliable and robust than optimizing dry residue. This is also remarkable as rosmarinic acid is a highly bioactive compound, while most of the molecules obtained in the dry residue might not have relevant bioactive properties.

To validate the predictive accuracy of the response surface methodology (RSM), independent extractions were conducted under the optimized conditions for both HAE and UAE (Table 2). The resulting extracts were subsequently analyzed using HPLC-DAD-ESI/MS/MS for the quantification of rosmarinic acid. In the case of HAE, the experimentally confirmed concentration of rosmarinic acid was 72.07 mg/g E, which closely matched the predicted value of 72.6 mg/g E, yielding a relative error of 0.83%. Similarly, for UAE, the experimentally obtained concentration was 73.6 mg/g E, compared to the predicted 73.5 mg/g E, corresponding to a relative error of 0.14%. These results reinforce the robustness and predictive reliability of the RSM models and support their effectiveness in maximizing the recovery of bioactive compounds. The quantified levels of rosmarinic acid and other phenolic constituents are presented in Table 2.

### 3.2. Bioactivity Properties

For each of the extraction techniques used, HAE and UAE, the optimal extracts enriched in rosmarinic acid were further analyzed for their bioactivity, which included antioxidant, anti-inflammatory, and antimicrobial activities.

#### 3.2.1. Antioxidant Activity

Table 3 summarizes the results obtained in the various in vitro assays, showing that both the selected UAE and HAE extracts displayed potent antioxidant activity, but distinct differences were observed between the methods.

In the TBARS assay, the extract of UAE presented a lower IC_50_ (8.9 µg/g compared to 12.8 µg/g), suggesting a greater capacity to inhibit oxidative damage in lipid-based systems. UAE’s superior performance in the lipid peroxidation assay can be attributed to its higher content of rosmarinic acid hexoside and luteolin-7-O-glucuronide. These compounds are potent inhibitors of oxidative damage, particularly in lipid-based systems, and their preservation is favored by UAE’s milder conditions, which reduce the risk of thermal degradation. Both extracts showed remarkably lower IC_50_ values than the antioxidant additives E-223 (sodium metabisulphite) and E-302 (calcium ascorbate). Additionally, better antioxidant activity was obtained compared to Trolox, a water-soluble vitamin E analogue widely used as a positive control in antioxidant biochemical tests. In contrast, the HAE extract demonstrated superior capacity in the reducing power and DPPH radical scavenging assays, likely attributed to its higher concentration of gallocatechin. This compound is known for its strong electron-donating properties due to its multiple hydroxyl groups, which also increases its hydrophilicity and makes it particularly effective in these assays. Notably, in the reducing power assay, HAE achieved an EC_50_ of 16.5 µg/mL, which is nearly 2.5 times lower than that of UAE (40.4 µg/mL).

These results indicate that HAE conditions are particularly effective in favoring the extraction of compounds with robust reducing potential, thereby significantly enhancing the antioxidant capacity. Although the samples are not identical, a comparison with the findings by Martins et al. [32], who also evaluated the antioxidant activity of *T. vulgaris* extracts obtained by agitation at 25 °C with 80% methanol, hot water (infusions), and boiling water (decoction), shows that the EC_50_ values obtained in the present study for both extraction methods were slightly lower, indicating that the optimization process may improve the free radical scavenging potential. When compared to the study by Habashy et al. [7], which evaluated the antioxidant activity of aqueous *T. vulgaris* extracts obtained by agitation at 25 °C during 24 h, the optimized UAE and HAE conditions resulted in significantly lower EC_50_ values across all assays, including lipid peroxidation, reducing power, and DPPH radical scavenging, which further underscores the effectiveness of the optimization process in enhancing the antioxidant potential of *T. vulgaris* extracts.

Regarding the OxHLIA assay, both extracts showed significant anti-haemolytic effects, but UAE performed slightly better in extending the protection time against haemolysis, possibly due to its luteolin derivatives, which are potent stabilizers of red blood cell membranes [33]. HAE and UAE extracts obtained IC_50_ values lower when compared with Trolox, a water-soluble vitamin E analogue widely used as a control antioxidant in biochemical tests, and lower than the commercial food grade antioxidant, E-223. E-302, a synthetic additive used as an antioxidant, preservative, and disinfectant (EFSA, 2018), did not evidence anti-haemolytic effects in the concentration range tested. This lack of activity may be attributed to the potential of E-302 to induce red blood cell (RBC) sickling, compromising membrane integrity and leading to haemolysis, as reported in previous studies [34,35]. In terms of cellular antioxidant activity (CAA), the HAE extract demonstrated a higher inhibition percentage than UAE—37% and 31%, respectively. It is worth mentioning that no information regarding the antioxidant activity of thyme extracts evaluated by this methodology was found in the literature.

The antioxidant activity observed across the different assays is likely a result of the synergistic action of multiple phenolic compounds. While rosmarinic acid was the most abundant, other phenolics may also contribute to the overall bioactivity. For example, caffeic acid, a hydroxycinnamic acid structurally related to rosmarinic acid, is known for its hydrogen-donating ability and lipid peroxidation inhibition. Luteolin-7-*O*-glucoside and apigenin derivatives, although present in lower amounts, possess established antioxidant properties through radical scavenging and modulation of oxidative stress pathways [36,37]. The complexity of the extract suggests that the antioxidant effects cannot be only attributed to rosmarinic acid but rather to a collective interaction of structurally diverse phenolics.

#### 3.2.2. Anti-Inflammatory and Antiproliferative Activity

The anti-inflammatory activity results are presented in Table 3, evidencing that the UAE extract demonstrated superior performance, with a lower IC_50_ value (67.7 µg/mL) compared to HAE (89.4 µg/mL) in RAW 264.7 cells. This can be attributed to UAE’s enhanced recovery of luteolin-7-O-glucuronide and caffeic acid derivatives, which are well-documented inhibitors of pro-inflammatory mediators [38]. HAE, despite its slightly lower anti-inflammatory activity, benefits from its higher gallocatechin content. This compound has shown potential anti-inflammatory effects, although its contribution appears less pronounced than that of the compounds enriched in the UAE extract.

Oliveira et al. [39] reported that the concentration of *T. vulgaris* extract from 25 to 100 mg/mL promotes the cell viability of RAW 264.7 cells. In the present study, the IC_50_ results obtained for the HAE and UAE extracts varied between 67.7 ± 0.3 and 89.4 ± 0.2 μg/mL, representing a significant improvement compared to previous studies (IC_50_ values approximately 1000 times lower), highlighting the efficiency of the rosmarinic-acid-enriched extracts in enhancing the anti-inflammatory activity.

Table 4 also provides cytotoxicity data, demonstrating that the *T. vulgaris* extracts rich in rosmarinic acid exhibited cytotoxic effects on the tested tumor cell lines. Overall, the exhibited cytotoxicity agrees with the literature [31]. Although cytotoxicity was also observed in non-tumoral VERO cells, the concentrations required to achieve anti-inflammatory and antioxidant effects were lower than those associated with cytotoxic activity in VERO cells for both extracts. Moreover, considering an intake of 200 mg of extract by a 70 kg adult with an average blood volume of 5L, the estimated systemic distribution would be lower than the determined IG_50_ in VERO cells. However, to estimate a safe intake level of these extracts, further studies would be required, including on bioavailability and long-term exposure.

The Selectivity Index (SI) is a crucial parameter for assessing the cytotoxic selectivity of bioactive compounds, calculated as the ratio between the IC_50_ values for normal cells and cancer cells. An SI value greater than 2 is generally considered indicative of strong selectivity towards cancer cells, making the extract a promising candidate for therapeutic applications. In this study, both UAE and HAE thyme extracts exhibited moderate selectivity, with SI values close to or slightly above 1. This suggests that while the extracts have some preference for targeting cancer cells over normal cells, their selectivity is not pronounced.

These findings are consistent with previous studies investigating the impact of different extraction methods on the phenolic compound profile and their biological activity. For instance, research on the effects of gamma irradiation on *Thymus vulgaris* and *Mentha x piperita* demonstrated that processing method alterations can influence phenolic composition and extract cytotoxicity [40].

The superior anti-inflammatory activity observed in UAE extracts can be partly attributed to the higher concentrations of luteolin-7-O-glucuronide and caffeic acid derivatives. Luteolin-7-O-glucuronide has been shown to inhibit inflammatory responses by downregulating key mediators such as nitric oxide (NO), inducible nitric oxide synthase (iNOS), and pro-inflammatory cytokines (IL-6, IL-1β, TNF-α) through suppression of the TAK1/NF-κB and MAPK signaling pathways [38,41]. Similarly, caffeic acid derivatives have demonstrated the ability to attenuate inflammation by modulating the Nrf2/HO-1 antioxidant pathway and reducing oxidative stress-mediated inflammatory cascades [42].

To enhance the specificity and efficacy of these extracts, further purification or fractionation could be employed. Such processes can concentrate the active compounds responsible for anticancer activity, thereby increasing the SI and reducing potential toxicity to normal cells.

#### 3.2.3. Antimicrobial Activity

Table 5 provides a comparison of the antimicrobial activities of the UAE and HAE optimal extracts enriched with rosmarinic acid. Overall, the UAE extract exhibited superior bactericidal and fungicidal activity, with lower MIC and MBC/MFC values across most tested strains. For example, against *E. coli* and *L. monocytogenes*, UAE achieved MICs of 0.25 mg/mL, compared to 1 mg/mL for HAE. When compared with previous data reported by Spréa et al. [31], who evaluated the antimicrobial activity of a *T. vulgaris* hydroethanolic (80%) extract obtained by maceration for 1 h at room temperature with a plant:solvent ratio of 40 g/L, a better activity was observed for the enriched UAE extract against *S. aureus*, *L. monocytogenes, E. coli*, *S. Typhimurium* and *E. cloacae*. On the contrary, the HAE-enriched extract only showed improved activity against *E. coli* and *E. cloacae*, while showing higher MIC and MBC values against *B. cereus* and *L. monocytogenes*. Therefore, the superior antibacterial activity of the UAE extract is likely due to the enhanced extraction of rosmarinic acid hexoside, caffeic acid derivatives, and luteolin-7-*O*-glucuronide in the UAE extract, which may act synergistically with the higher rosmarinic acid content resulting in more potent antibacterial properties through membrane disruption and enzymatic inhibition. Moreover, the difference in solvent concentration (27% and 50% for HAE and UAE, respectively), may also favor the co-extraction of other compounds that contributed to different antimicrobial effects. For comparison, commercial food additives, namely sodium benzoate (E211) and potassium metabisulphite (E224), were tested as positive controls. Compared to both UAE and HAE extracts enriched with rosmarinic acid, only E211 demonstrated superior activity against *B. cereus*.

Similarly, for the antifungal activity (Table 5), the UAE extract showed lower MIC and MFC values than the HAE extract, suggesting superior efficacy against the panel of selected microorganisms. Contrary to what was observed for the antibacterial activity, compared to the results previously obtained by Spréa et al. [31], both extracts enriched with rosmarinic acid exhibited similar or lower antifungal activity. This suggests that rosmarinic acid is unlikely to be the primary component responsible for the antifungal activity. Potassium metabisulphite (E224), a food additive with antimicrobial properties, demonstrated superior MIC and MFC values compared to the HAE extract across all tested fungi, as the HAE extract consistently showed higher MIC and MFC values, indicating poorer performance. The UAE extract, however, outperformed both E224 and HAE in most cases, except for *P. funiculosum* and *T. viride,* for which E224 achieved lower MIC.

Moreover, the enhanced bactericidal and fungicidal activities of UAE extracts are likely a result of the synergistic action of rosmarinic acid, luteolin derivatives, and caffeic-acid-related compounds. Rosmarinic acid has been reported to cause structural damage to microbial membranes, leading to increased permeability and cellular content leakage [43,44]. Luteolin and its derivatives further contribute to antimicrobial efficacy by disrupting bacterial membrane integrity and inhibiting biofilm formation [45]. Additionally, caffeic acid derivatives destabilize microbial membranes and interfere with critical metabolic functions, enhancing the overall antimicrobial effect.

## 4. Conclusions

This study successfully optimized the extraction of bioactive compounds from *Thymus vulgaris* L., utilizing both HAE and UAE techniques. The RSM-predicted optimal extraction conditions for rosmarinic acid that were experimentally validated in this study. The optimization focused primarily on maximizing rosmarinic acid content due to its significant antioxidant and anti-inflammatory properties, characteristic of Lamiaceae family species. Applying the Box–Behnken design, optimal extraction conditions were established for both methods, allowing to obtain extracts enriched in rosmarinic acid and potentially exhibiting higher bioactivity. Overall, UAE produced extracts with superior bioactivity and requiring a shorter extraction time and lower solvent consumption, suggesting that this method could be more effective and sustainable for industrial applications. However, its large-scale feasibility requires further evaluation considering equipment cost, solvent usage, energy consumption, maintenance, and industrial adaptability. In contrast, although HAE may require higher solvent volumes and longer processing times, its simplicity and adaptability to existing extraction set-ups could be advantageous for large-scale applications. Regarding solvent consumption, both methods employed hydroethanolic solutions with relatively low ethanol concentrations (27–50%), which can be favorable from an environmental and economic perspective when solvent recovery is not performed. Nevertheless, at industrial scale, solvent recovery by standard distillation systems is typically implemented to enhance process sustainability. In this context, the energy expenditure to recover the solvent should also be considered since lower ethanol concentrations may increase energy demands for solvent recovery, potentially affecting overall process efficiency. Therefore, further studies should include a life cycle assessment to fully assess the energy expenditure, large-scale feasibility, and overall sustainability of both processes. The results support the use of *T. vulgaris* as a valuable natural resource, with optimized extraction parameters enhancing its potential as a functional ingredient.

However, it is important to acknowledge that this study was conducted using in vitro models only, which may not fully represent the biological complexity of in vivo systems. Future studies incorporating in vivo models are recommended to better assess the bioavailability and long-term efficacy of the extracted compounds, as well as the long-term stability of the extract.

In conclusion, the optimized extraction protocols developed here align with the growing demand for sustainable and efficient methodologies, offering the food and pharmaceutical industries viable alternatives to synthetic additives while promoting the use of natural bioactive compounds.

## Figures and Tables

**Table 1:** Experimental variables tested, the intervals and the coding for calculating the design distribution BBD with three factors.

	HAE			UAE		
	*t* (min)	*T* (°C)	*S* (%)	*t* (min)	*P* (W)	*S* (%)
−1	5	25	0	5	50	0
0	92.5	62.5	50	32.5	275	50
1	180	100	100	60	500	100

**Table 2:** Experimental design applied in the RSM analysis of the BBD for heat and ultrasound-assisted-extraction (HAE and UAE) of *Thymus vulgaris* L., encoded and natural values, and responses obtained (extraction yield, phenolic compounds, and optimal points generated). Results are expressed in mean ± sd (mg/g of extract).

Run	Experimental Design						Responses									
	Coded Values			Natural Values			Extract	Phenolic Compounds by HPLC-DAD-ESI-MS/MS								TPC
	X_1_	X_2_	X_3_	t	T/*P*	S	E	P1	P2	P3	P4	P5	P6	P7	P8	
				(min)	(°C/W)	(%)	(g)	(mg/g E)	(mg/g E)	(mg/g E)	(mg/g E)	(mg/g E)	(mg/g E)	(mg/g E)	(mg/g E)	(mg/g E)
Heat-assisted-extraction																
1	0	1	1	92.5	100	100	0.013	9.6 ± 0.1	0.9 ± 0.1	*nd*	*nd*	*nd*	0.2 ± 0.1	8.4 ± 0.5	1.3 ± 0.1	20.4 ± 0.7
2	0	1	−1	92.5	100	0	0.022	49.4 ± 1.9	6.7 ± 0.3	4.2 ± 0.1	0.11 ± 0.01	15.8 ± 0.9	7.2 ± 0.3	68.7 ± 4.1	0.2 ± 0.1	152.3 ± 7.6
3	0	−1	1	92.5	25	100	0.005	13.3 ± 0.1	1.3 ± 0	2.4 ± 0.1	0.11 ± 0.01	4.0 ± 1.0	1.7 ± 0.1	16.0 ± 0.1	0.6 ± 0.1	39.4 ± 0.1
4	0	−1	−1	92.5	25	0	0.01	42.6 ± 0.9	2.8 ± 0.1	1.7 ± 0.1	*nd*	4.4 ± 0.1	0.4 ± 0.1	2.7 ± 0.1	0.3 ± 0.1	54.8 ± 1.1
5	1	0	1	180	62.5	100	0.011	5.3 ± 0.4	*nd*	*nd*	*nd*	*nd*	0.2 ± 0.1	6.1 ± 0.1	0.7 ± 0.1	12.2 ± 0.5
6	1	0	−1	180	62.5	0	0.015	32.7 ± 1.5	8.0 ± 0.1	5.1 ± 0.2	0.11 ± 0.01	9.5 ± 0.5	0.7 ± 0.1	23.0 ± 1.0	0.6 ± 0.1	79.2 ± 3.4
7	−1	0	1	5	62.5	100	0.006	2.8 ± 0.1	*nd*	*nd*	*nd*	*nd*	*nd*	2.1 ± 0.1	0.3 ± 0.1	5.2 ± 0.1
8	−1	0	−1	5	62.5	0	0.005	45.5 ± 0.5	1.8 ± 0.1	1.6 ± 0	*nd*	2.1 ± 0.1	0.3 ± 0.1	2.5 ± 0.2	0.2 ± 0.1	53.9 ± 0.9
9	1	1	0	180	100	50	0.023	24.0 ± 1.0	6.4 ± 0.3	9.5 ± 0.5	1.1 ± 0.1	6.5 ± 0.4	6.9 ± 0.3	71.7 ± 3.7	1.3 ± 0.1	127.7 ± 6.3
10	1	−1	0	180	25	50	0.002	5.3 ± 0.1	*nd*	*nd*	*nd*	*nd*	0.2 ± 0.1	6.1 ± 0.7	0.7 ± 0.1	12.2 ± 1
11	−1	1	0	5	100	50	0.034	10.7 ± 0.4	1.1 ± 0.1	2.3 ± 0.2	0.2 ± 0.1	3.7 ± 0.2	1.6 ± 0.1	21.4 ± 0.8	1.3 ± 0.1	42.3 ± 1.7
12	−1	−1	0	5	25	50	0.002	2.1 ± 0.1	0.6 ± 0.1	*nd*	*nd*	*nd*	0.11 ± 0.01	3.4 ± 0.1	*nd*	6.1 ± 0.2
13	0	0	0	92.5	62.5	50	0.008	16.7 ± 0.6	3.6 ± 0.2	5.5 ± 0.3	0.4 ± 0.1	6.0 ± 1.0	3.8 ± 0.1	56.0 ± 1.0	1.4 ± 0.1	93.4 ± 2.6
14	0	0	0	92.5	62.5	50	0.003	18.0 ± 1.0	2.4 ± 0.1	6.3 ± 0.2	0.4 ± 0.1	4.4 ± 0.2	4.2 ± 0.2	47.0 ± 3.0	1.1 ± 0.1	83.7 ± 3.8
15	0	0	0	92.5	62.5	50	0.013	22.0 ± 1.0	2.3 ± 0.1	6.4 ± 0.2	0.4 ± 0.1	5.9 ± 0.1	4.4 ± 0.1	54.8 ± 2.6	1.0 ± 0.1	97.4 ± 4.4
16	0	0	0	92.5	62.5	50	0.017	33.0 ± 1.0	2.6 ± 0.2	2.9 ± 0.1	0.6 ± 0.1	6.5 ± 0.3	5.9 ± 0.3	72.6 ± 3.7	1.5 ± 0.1	124.9 ± 5.6
17	0	0	0	92.5	62.5	50	0.013	17.8 ± 0.4	2.5 ± 0.1	4.5 ± 0.1	0.3 ± 0.1	9.6 ± 0.3	3.7 ± 0.1	44.2 ± 1.7	1.1 ± 0.1	83.5 ± 2.7
Ultrasound-assisted-extraction																
1	0	1	1	32.5	500	100	0.013	8.6 ± 0.3	1.1 ± 0.1	*nd*	*nd*	1.2 ± 0.1	0.2 ± 0.1	4.8 ± 0.1	0.9 ± 0.1	16.8 ± 0.5
2	0	1	−1	32.5	500	0	0.022	16.4 ± 0.7	3.6 ± 0.1	2.4 ± 0.1	0.15 ± 0.01	5.1 ± 0.1	0.15 ± 0.01	4.4 ± 0.1	0.6 ± 0.1	32.6 ± 0.9
3	0	−1	1	32.5	50	100	0.005	1.1 ± 0.1	0.5 ± 0.1	*nd*	*nd*	*nd*	*nd*	0.8 ± 0.1	*nd*	2.4 ± 0.1
4	0	−1	−1	32.5	50	0	0.01	4.7 ± 0.1	0.7 ± 0.1	*nd*	*nd*	*nd*	*nd*	1.4 ± 0.1	*nd*	6.8 ± 0.1
5	1	0	1	60	275	100	0.011	2.6 ± 0.1	0.6 ± 0.1	*nd*	*nd*	*nd*	*nd*	1.6 ± 0.1	0.2 ± 0.1	5.1 ± 0.1
6	1	0	−1	60	275	0	0.015	8.3 ± 0.1	1.7 ± 0.1	*nd*	*nd*	1.3 ± 0.1	0.1 ± 0.1	1.6 ± 0.1	0.15 ± 0.01	14.1 ± 0.2
7	−1	0	1	5	275	100	0.006	1.5 ± 0.1	0.5 ± 0.1	*nd*	*nd*	*nd*	*nd*	0.7 ± 0.1	*nd*	2.7 ± 0.1
8	−1	0	−1	5	275	0	0.005	1.6 ± 0.1	0.6 ± 0.1	*nd*	*nd*	*nd*	*nd*	0.7 ± 0.1	*nd*	2.9 ± 0.1
9	1	1	0	60	500	50	0.023	17.1 ± 0.6	3.9 ± 0.1	4.8 ± 0.1	0.8 ± 0.1	12.6 ± 0.4	8.8 ± 0.5	73.5 ± 1.6	1.4 ± 0.1	123 ± 3.3
10	1	−1	0	60	50	50	0.002	3.8 ± 0.1	0.7 ± 0.1	*nd*	*nd*	*nd*	0.2 ± 0.1	5.2 ± 0.1	*nd*	9.9 ± 0.1
11	−1	1	0	5	500	50	0.034	11.7 ± 0.1	1.2 ± 0.1	0.5 ± 0.1	0.15 ± 0.01	2.1 ± 0.1	1.1 ± 0.1	15.8 ± 0.2	0.3 ± 0.1	33.0 ± 0.4
12	−1	−1	0	5	50	50	0.002	1.0 ± 0.1	*nd*	*nd*	*nd*	*nd*	*nd*	1.2 ± 0.1	*nd*	2.2 ± 0.1
13	0	0	0	32.5	275	50	0.008	4.3 ± 0.1	0.8 ± 0.1	*nd*	*nd*	5 ± 0.3	0.5 ± 0.1	7.6 ± 0.2	0.15 ± 0.01	18.4 ± 0.6
14	0	0	0	32.5	275	50	0.003	5.9 ± 0.2	1.1 ± 0.1	*nd*	*nd*	1.3 ± 0.1	0.6 ± 0.1	8.9 ± 0.2	0.15 ± 0.01	18.0 ± 0.4
15	0	0	0	32.5	275	50	0.013	8.2 ± 0.1	0.9 ± 0.1	*nd*	*nd*	1.8 ± 0.1	1.1 ± 0.1	9.9 ± 0.4	0.15 ± 0.01	21.9 ± 0.6
16	0	0	0	32.5	275	50	0.017	3.6 ± 0.2	0.7 ± 0.1	*nd*	*nd*	1.6 ± 0.1	0.6 ± 0.1	7.6 ± 0.4	0.2 ± 0.1	14.2 ± 0.7
17	0	0	0	32.5	275	50	0.013	9.7 ± 0.3	1.1 ± 0.1	*nd*	0.15 ± 0.01	*nd*	0.8 ± 0.1	12.1 ± 0.4	0.2 ± 0.1	23.9 ± 0.7
Optimal values																
HAE	0	1	1	121	91	27	-	32.2 ± 0.6	2.6 ± 0.1	2.9 ± 0.1	0.6 ± 0	6.5 ± 0.3	5.9 ± 0.2	72 ± 2.9	1.5 ± 0.1	124.2 ± 3.3
UAE	1	1	0	60	500	50	-	17.1 ± 0.4	3.9 ± 0.1	4.8 ± 0.1	0.8 ± 0	12.7 ± 0.3	8.8 ± 0.3	73.6 ± 1.1	1.3 ± 0.1	123 ± 1.1

Phenolic Compounds: Gallocatechin (P1); Caffeic acid hexoside (P2); Caffeic acid acetylhexoside (P3); Apigenin-C-hexoside-O-hexoside (P4); Rosmarinic acid hexoside (P5); Luteolin−7-O-glucuronide (P6); cis-Rosmarinic acid (P7); Luteolin-O-diglucuronide (P8); Non-detected (nd); Total Phenolic Compounds (TPC). Calibration curves: Apigenin−6−*C*−glucoside y = 225,142x + 112,350, R^2^ = 0.9989 (P4, P6 and P8); Caffeic acid y = 47,221x − 228,640, R^2^ = 0.9989 (P2 and P3); Catechin y = 8387,1x − 71124 (P1), R^2^ = 0.9964; Rosmarinic acid y = 25,054x − 171,281, R^2^ = 0.9975 (P5 and P7).

**Table 3:** Antioxidant activity of *T. vulgaris* extracts obtained under optimal ultrasound-assisted extraction (UAE) and heat-assisted extraction (HAE) conditions.

	TBARS ^1^	Reducing Power ^2^	DPPH ^2^	OxHLIA ^1^		CAA ^3^
				Δt = 60 min	Δt = 120 min	
UAE	8.9 ± 0.1 ^a^	40.4 ± 0.1 ^a^	283 ± 20 ^a^	1.4 ± 0.1 ^a^	6.6 ± 0.3 ^a^	31 ± 1%
HAE	12.8 ± 0.5 ^a^	16.5 ± 0.1 ^b^	108 ± 4 ^b^	5.6 ± 0.3 ^b^	23 ± 2 ^b^	37 ± 3%
E-223	228.7 ± 0.1 ^b^	53.5 ± 2.5 ^a^	43 ± 1 ^c^	41 ± 1 ^c^	84 ± 2 ^c^	-
E-302	284.0 ± 0.1 ^b^	8.8 ± 0.4 ^c^	21 ± 3 ^d^	-	-	-
Trolox	23.0 ± 0.1 ^c^	13.6 ± 0.1 ^b^	22.7 ± 1.2 ^d^	21.8 ± 0.2 ^d^	43.5 ± 0.3 ^d^	-
Quercetin	-	-	-	-	-	95 ± 5%

^1^: results expressed in IC_50_ (μg/mL); ^2^: results expressed in EC_50_ (μg/mL); ^3^: maximum concentration tested: 2000μg/mL. E-223: Sodium metabisulfite; E-302: Calcium ascorbate. Different letters in the same row indicate significant differences (*p* < 0.05) between samples.

**Table 4:** Antiproliferative potential and selectivity index (SI) of *T. vulgaris* extracts obtained under optimal UAE and HAE conditions. Different letters in the same row indicate significant differences (*p* < 0.05) between samples. Human gastric epithelial cell line (AGS), human colorectal adenocarcinoma (CaCo2), breast carcinoma (MCF-7), and kidney epithelial cells extracted from an African green monkey (VERO).

Cell Line	Cytotoxicity/Anti-Inflammatory Activity (GI_50_ Values; µg/mL)				Selectivity Index (Mean ± SD)	
	HAE	UAE	Ellipticine	Dexamethasone	sHAE	UAE
AGS	139 ± 5 ^a^	133 ± 10 ^a^	1.23 ± 0.03	-	0.80 ± 0.06	0.61 ± 0.05
CaCo2	247 ± 23 ^b^	263 ± 15 ^b^	1.21 ± 0.03	-	1.46 ± 0.07	1.19 ± 0.06
MCF-7	254 ± 7 ^c^	232 ± 5 ^c^	1.02 ± 0.02	-	1.51 ± 0.06	1.10 ± 0.04
VERO	172 ± 3 ^f^	219 ± 16 ^f^	1.29 ± 0.02	-	-	-
RAW 264.7	89.4 ± 0.2 ^g^	67.7 ± 0.3 ^g^	-	5.97 ± 0.84	-	-

**Table 5:** Antibacterial and antifungal activity of *T. vulgaris* extracts (mg/mL) obtained under optimal UAE and HAE conditions.

Microbial Strain	HAE		UAE		E211		E224	
**Antibacterial activity**								
	MIC	MBC	MIC	MBC	MIC	MBC	MIC	MBC
*Staphylococcus aureus*	1	2	0.5	1	4	4	1	1
*Bacillus cereus*	0.5	1	0.25	0.5	0.5	0.5	2	4
*Listeria monocytogenes*	1	2	0.25	0.5	1	2	0.5	1
*Escherichia coli*	0.25	0.5	0.25	0.5	1	2	0.5	1
*Salmonella typhimurium*	1	2	0.5	1	1	2	1	1
*Enterobacter cloacae*	0.5	1	0.25	0.5	2	4	0.5	0.5
**Antifungal activity**								
	MIC	MFC	MIC	MFC	MIC	MFC	MIC	MFC
*Aspergillus fumigatus*	2	4	0.5	1	1	2	1	1
*Aspergillus niger*	1	2	0.5	1	1	2	1	1
*Aspergillus versicolor*	2	4	1	2	2	2	1	1
*Penicillium funiculosum*	2	4	1	2	1	2	0.5	0.5
*Penicillium verrucosum var. cyclopium*	1	2	0.5	1	2	4	1	1
*Trichoderma viride*	2	4	2	4	1	2	0.5	0.5

Abbreviations: MIC: minimum inhibitory concentration; MBC; minimum bactericidal concentration; MFC: minimum fungicidal concentration; E211: sodium benzoate; E224: potassium metabisulphite.

## Data Availability

The original contributions presented in this study are included in the article/Supplementary Materials. Further inquiries can be directed to the corresponding author.

## Supplementary Materials

The following supporting information can be downloaded at: https://www.mdpi.com/article/10.3390/antiox14050567/s1, Table S1: Identification of phenolic compounds in *Thymus vulgaris* extracts based on their retention time, UV absorbance maxima (λmax), and mass spectrometry fragmentation patterns ([M-H]^−^ and MS^2^); Figure S1: Contour plots of the dry residue (*R_HAE_*) for HAE and UAE; Figure S2: Contour plots of rosmarinic acid (*RA_HAE_*) for HAE and UAE.

## Abbreviations

The following abbreviations are used in this manuscript:

CAA	Cellular Antioxidant Activity
DPPH	2,2-diphenyl-1-picrylhydrazyl
HAE	Heat-assisted extraction
MBC	Minimum bactericidal concentration
MFC	Minimum fungicidal concentration
MIC	Minimum inhibitory concentration
OxHLIA	Oxidative Hemolysis Inhibition Assay
RSM	Response surface methodology
SD	Standard deviation
TBARS	Thiobarbituric Acid Reactive Substances
TPC	Total phenolic content
UAE	Ultrasound-assisted extraction
