# Supplementary material for: Effect of Heat-Assisted and Ultrasound-Assisted Extraction Methods on the Phenolic Profile and Biological Activity of Thymus vulgaris L. Extracts
Source: Antioxidants (Basel). 2025 May 9;14(5):567. doi: 10.3390/antiox14050567 (PMC12108244; doi:10.3390/antiox14050567)
Supplement: Supplementary file 1 [file antioxidants-14-00567-s001.zip › antioxidants-3532113-supplementary.pdf]

Supplementary Material

Effect of Heat-Assisted and Ultrasound-Assisted Extraction Methods on the Phenolic Profile and Biological Activity of *Thymus vulgaris* L. extracts

Rafael Mascoloti Spréa[1,2], Cristina Caleja[1], Eliana Pereira[1*], Márcio Carocho[1], José Pinela[1,3], Tiane C. Finimundy[1], Ricardo C. Calhelha[1], Marina Kostić[4], Marina Sokovic[4], Miguel A. Prieto[2], Joana S. Amaral[1], Lillian Barros[1]

Table S1. Identification of phenolic compounds in *Thymus vulgaris* extracts based on their retention time, UV absorbance maxima (λmax), and mass spectrometry fragmentation patterns ([M-H]⁻ and MS²).

Peak	Retention Time (min)	λ_{max}	[M-H]⁻ *m/z*	MS2
**Gallocatechin**	4.338	263	305	219(45). 179(41). 125(100)
**Caffeic acid hexoside**	4.978	324	341	179(100)
**Caffeic acid acetylhexoside**	6.308	315	387	369(25). 207(100). 163(47)
**Apigenin-*C*-hexoside-*O*-hexoside**	8.956	321	593	503(21). 473(100). 383(17). 353(32)
**Rosmarinic acid hexoside**	18.173	322	521	359(100)
**Luteolin-7-*O*-glucuronide**	19.659	343	461	285(100)
**cis-Rosmarinic acid**	20.887	328	359	197(15). 179(21). 161(100)
**Luteolin-*O*-diglucuronide**	27.396	328	637	351(6). 285(5). 283(100)

Figure S1. Contour plots of the dry residue (R_{HAE}) for HAE and UAE.

Extract (g)	
HAE	UAE
A1	B1

A1

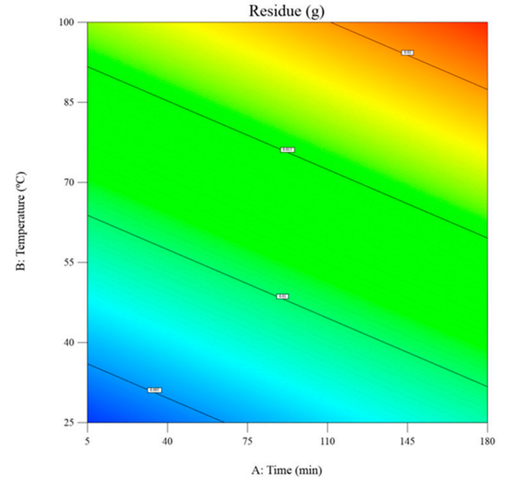

B1

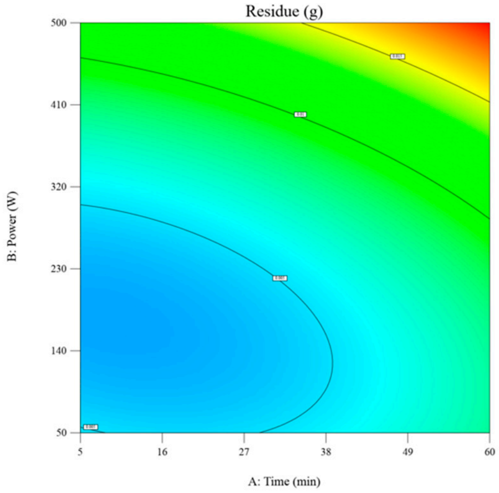

A2

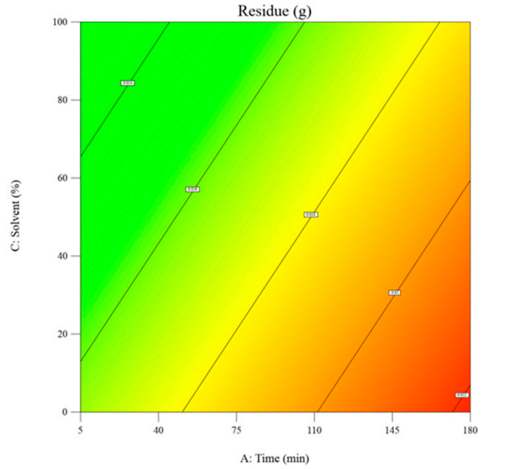

B2

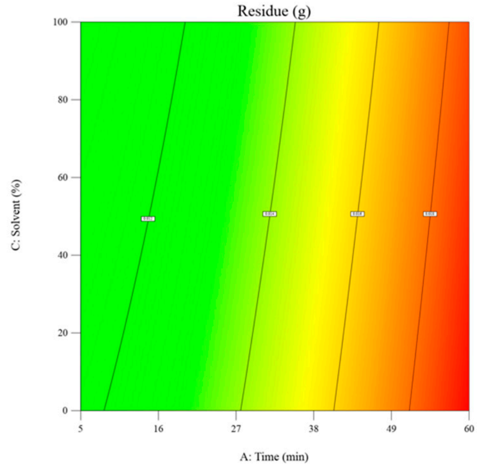

A3

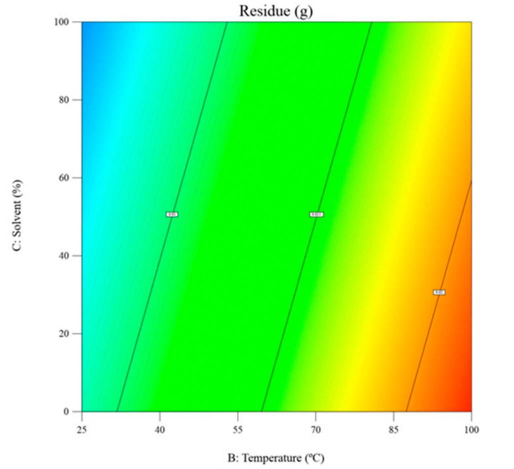

B3

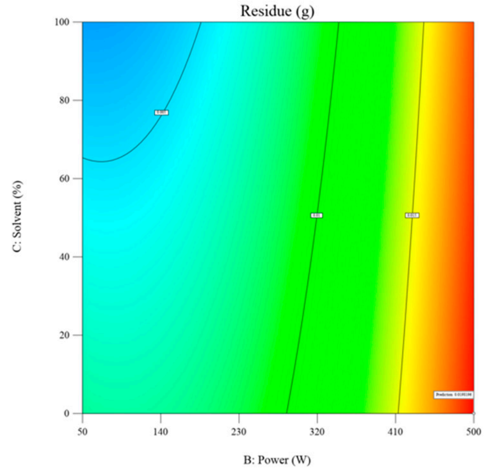

Figure S2. Contour plots of rosmarinic acid (RA_{HAE}) for HAE and UAE.

Rosmarinic Acid (mg/g E)	
HAE	UAE
A1<br>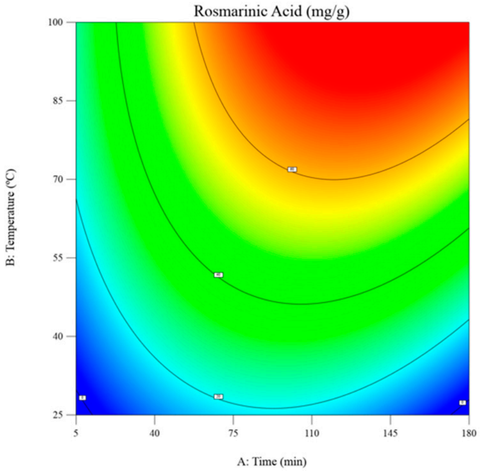 	B1<br>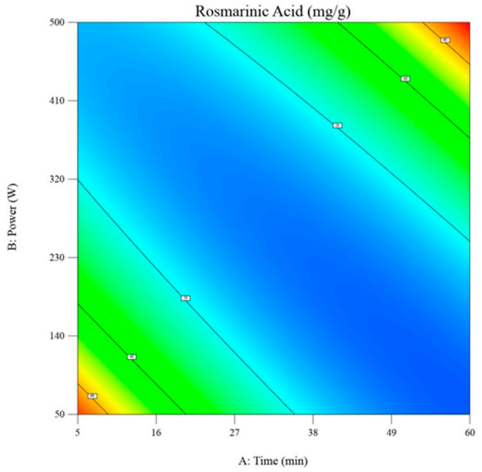
A2<br>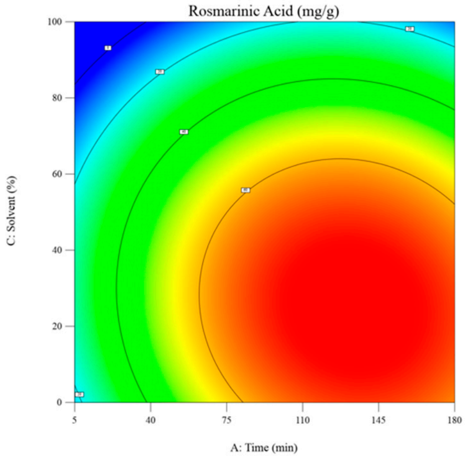 	B2<br>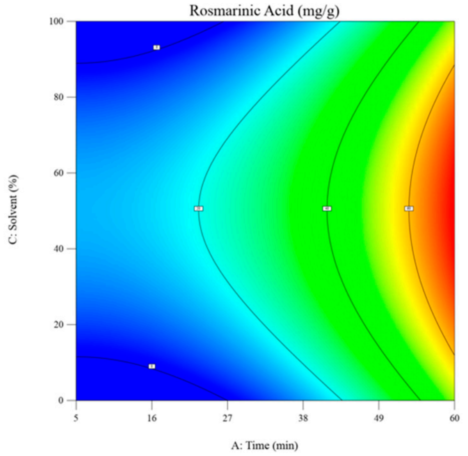
A3<br>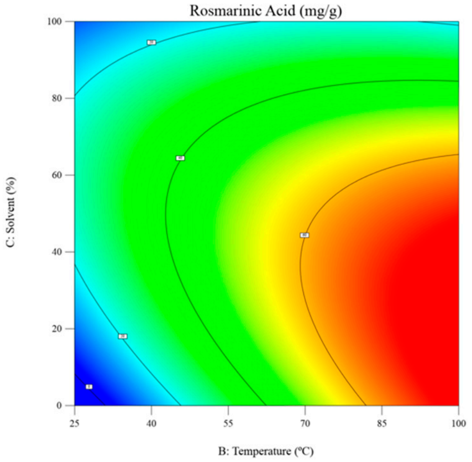 	B3<br>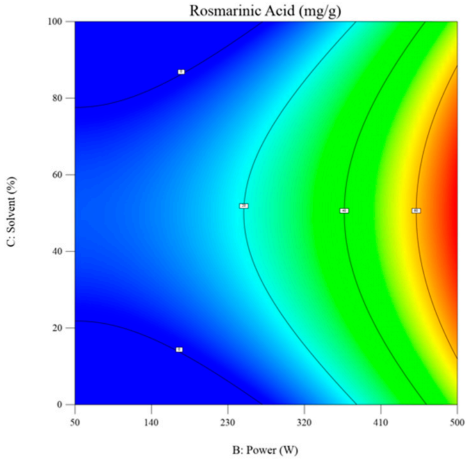

Figure S1 and S2 represent the contour plots of the two extraction types, heat-assisted extraction (HAE) and ultrasound-assisted extraction (UAE) for the dry residue (g) and rosmarinic acid (mg/g), respectively.

Comparing the two plots side by side in Figure S1 and considering the energy of the HAE is provided through heat and in UAE through ultrasonic waves, some conclusions can be extracted. The first row, plotting time against temperature (A1) and ultrasound power (B1), there are some similarities in which longer extraction time promote higher residue yield, as does high energy, shown by the red zones being at the top right of both plots. Still, while A1 is dominated by linear interactions, in UAE (B1, Figure S3) there are curved lines, suggesting a non-linear interaction. Overall, the better residue yields are shown at 85 ºC for A1, while in the UAE the orange zone is found very near the maximum of 500 W. Between these two plots, the behavior in terms of solid residue is very similar, a tendency to increase over time and with higher energy input either through temperature or ultrasonic waves.

Row 2 of Figure S2 shows the plots of time and solvent (A2, B2). For both extraction times, longer extractive periods promote higher yields, although there is a slight difference, in which the UAE shows low variation along solvent concentration, meaning that the interaction between time and ethanol was not very promoting of compounds' extraction. This is highlighted by a vertical column of orange and red colors, where the extraction is the highest. For HAE, very low solvent concentration favors the yield. For both plots the interaction was linear. Finally, in the last row, which plots solvent and energy, in the form of temperature for HAE and ultrasonic waves for UAE, a similar behavior was observed, namely a linear interaction at higher extraction temperature and ultrasound power, while at lower values the UAE showed nonlinear interaction, shown by circular lines in B3. Still, once again, solvent for UAE did not show much difference, while for HAE lower amounts favored extractability.

Overall, the behavior for both extraction types for residue yield was quite similar, with higher energy and extraction time favoring the yield, and the solvent playing a complete secondary role, especially in UAE, where the variation of ethanol did not have impact.

In Figure S3, the influence of extraction parameters on the yield of rosmarinic acid (mg/g E) is shown for both UAE and MAE across three factor combinations. Figures A1 and B1 plotting time and energy (temperature for HAE and ultrasound power for UAE), show that for HAE there is a nonlinear interaction, which favored the rosmarinic acid

extraction at 120 minutes and between 90 to 100 ºC; however, for UAE there was a linear interaction, although the behavior of these two factors was quite different. In this case, there are two zones with higher extractability, namely very low extraction times and low power, and high extraction times with high ultrasound power. This suggests that increasing extraction time or power tends to degrade rosmarinic acid. Still, at very high power and 60 minutes of extraction time there is an increase in this polyphenol. The second row, which examines the interaction between extraction time and solvent concentration, shows a more similar effect between both extraction types, with a nonlinear interaction. The similarity is mainly in longer extraction times, although HAE sets the highest extraction at about 130 minutes and UAE at 55 minutes (upper limit). The difference between these two is mainly on the influence of solvent concentration, in which for HAE 20 to 40% of ethanol reveal the highest yield, while for UAE, there is no significant difference between 20 to 80%, meaning a low influence of this factor for the overall yield. Finally, in the third row, which plots solvent and energy (temperature through heat for HAE and ultrasonic power for UAE), once again, higher values of energy promote a higher yield in rosmarinic acid, and, once again, ethanol between 0 and 60% show high yields for HAE, in UAE the interval extends from 15 to 95%, showing a secondary role. In both extraction types, the interaction was also nonlinear.

Overall, and considering both extraction methodologies and the two responses (dry residue and rosmarinic acid), ethanol has a very secondary role, being the least important factor. Long extraction times and high energy (heat or ultrasound) also promote the two responses.
